# The collaborative and professional boundary challenges from a bottom-up perspective: an insider action research study on a hospital ward

**DOI:** 10.1108/JHOM-03-2023-0093

**Published:** 2024-11-22

**Authors:** Mia Björk, Annika Eklund, Maria Skyvell Nilsson, Viola Nyman

**Affiliations:** Department of Health Sciences, University West, Trollhättan, Sweden; Department of Social and Behavioural Studies, University West, Trollhattan, Sweden; University of Gothenburg, Gothenburg, Sweden; Department of Research and Development, NU Hospital Group, Trollhattan, Sweden

**Keywords:** Interprofessional collaboration, Hospital resources, Insider action research, Professional boundaries, Professional responsibility

## Abstract

**Purpose:**

The aim of this study was to identify and describe the collaborative and professional boundary challenges at a hospital ward from a bottom-up perspective.

**Design/methodology/approach:**

The study was conducted as a bottom-up improvement project at a hospital ward in western Sweden. An insider action research (IAR) approach was used during the project. The theoretical framework for this project was based on the Cultural-Historical Activity Theory (CHAT). Data were collected between 2019 and 2021.

**Findings:**

The findings showed that unclear professional boundaries and limited resources challenged and hindered interprofessional collaboration. The project group had to reorganize its daily work to adjust to the different disciplines’ legal responsibilities in relation to the patients’ recovery process. To safely discharge patients, the professionals needed to talk about each other’s professional responsibilities, professional boundaries and ethical codes.

**Originality/value:**

The IAR project revealed that revising the daily team-round routine improved the status of assistant nurses and encouraged physicians to consider input from all professions during the patients’ recovery process. However, the new approach faced resistance from clinic leadership, who believed it could prolong patients’ stays in the ward. The findings underscore the challenges of modifying hierarchical structures and social orders within hospital settings.

## 1. Introduction

Contemporary hospital organizations face urgent challenges to balance limited resources with the public’s increased need for healthcare (Holmér *et al.*, 2023; Jones and Dolsten, 2024). Research has shown how limited personnel resources have challenged the professional roles, professional responsibilities and the hierarchy in healthcare teams (Richards *et al.*, 2000; Nancarrow and Borthwick, 2005). The present study examines a specific hospital ward in western Sweden that faced challenges due to the increasing public need for healthcare and the shortage of nurses. The ward conducted a yearly routine survey, which identified concerns about the ward’s patient safety; these concerns were related to the lack of nurses at the ward, unclear professional responsibilities and deficient collaboration between the professionals and external caregivers. Based on the results of the survey, the various professions at the ward were expected to work together on solutions to patient safety concerns. Specifically, there was a need to investigate how the collaboration between the professionals influenced the provision of safe care. McLaney *et al.* (2022) stated that many organizations define individual roles in interprofessional teams; however, adopting team-based skills could improve collaboration and care in complex hospital settings. Further, negotiating professional roles and responsibilities and reaching agreements on the optimal patient care approach from various professional perspectives have been shown to be crucial for effective collaboration within healthcare teams (Schot *et al.*, 2020). Thus, the aim of the present study was to identify and describe the collaborative and professional boundary challenges at a hospital ward from a bottom-up perspective. Insider action research (IAR) was used because it enables the researcher to be part of the improvement project and ongoing process, both during project meetings and in daily work. To analyze the complex improvement processes, the Cultural-Historical Activity Theory (CHAT) was used as a starting point (Engeström, 1999a, c). Using the CHAT can be valuable for understanding and describing the improvement processes within project groups. Building on the CHAT, the concept of expansive learning, developed by Engeström and Sannino (2020), was used to analyze the data. Expansive learning examines workplace collaboration and learning by focusing on the process itself, rather than having predefined goals. It looks at both expected and unexpected outcomes based on how professionals identify problems.

### 1.1 Background

Interprofessional collaboration has been described as essential to create a successful team and contribute to more efficient and sustainable healthcare organizations (Morley and Cashell, 2017; McNeil *et al.*, 2013; Shakhman *et al.*, 2020). Functional teamwork and collaboration have been shown to have positive effects on patient safety and healthcare quality (Morley and Cashell, 2017; McLaney *et al.*, 2022), and to avoid overcrowding hospitals (Hansson *et al.*, 2018; Blom, 2015; Hallgren *et al.*, 2021). More than a decade ago, the World Health Organization (WHO, 2010) highlighted the importance of developing healthcare workers’ interprofessional collaboration at the same pace as the medico-technical improvement in healthcare. However, several current challenges in healthcare have increased the demand for interprofessional collaboration (Richards *et al.*, 2000; Nancarrow and Borthwick, 2005). For example, studies have described a challenging balance between the increasing need for healthcare and an aging population (WHO, 2021). In addition, poor working conditions contribute to healthcare staff leaving their employment (Bahlman-van Oijen *et al*., 2023).

Healthcare organizations constantly innovate working approaches and processes to deliver high-quality care while managing available resources effectively (Morley and Cashell, 2017). Numerous initiatives have aimed to emphasize collaboration and enhance working methods among diverse healthcare professionals to improve overall system effectiveness, teamwork and patient care (Gadolin and Wikström, 2016). Van Tuyl *et al.* (2021) suggested that the concept of task-shifting holds promise for enhancing care quality and addressing evolving societal demands. Nevertheless, obstacles persist concerning the organizational adjustments accompanying task-shifting implementation. These challenges relate to shifts in professional hierarchies, professional boundaries and interprofessional teamwork dynamics (Feiring and Eidesvik Lie, 2018). Previous studies have demonstrated that redistributing tasks among hospital team members alters their roles and fundamental responsibilities, highlighting the importance of identifying such changes during reorganization efforts (Maier and Aiken, 2016). Hence, tasks that were previously “owned” by a specific profession can be performed by another profession; this challenges professional boundaries as the focus shifts from the professionals “owning” certain tasks to how to best use available competence (Nancarrow and Borthwick, 2005). Expanding professional boundaries within a team can also lead to challenging disputes between professionals over those boundaries. These disputes must be resolved to avoid hindering collaboration and consensus-making within the healthcare team (King *et al.*, 2015, Engeström and Pyörälä, 2021). However, different opinions across professional boundaries in a team could also serve as a catalyst for identifying necessary changes and improvements within the organization (Engeström and Pyörälä, 2021).

Moreover, recent research has shown that blurring or breaking down boundaries can have mixed or even negative consequences for collaboration (Farchi *et al.*, 2023; Langley *et al.*, 2019), which indicates that this is a paradoxical and complex knowledge field. This reasoning highlights the importance of further studies to clarify professional roles and boundaries in a hospital team to ensure a safe and qualitative collaboration in patient work (Richards *et al.*, 2000; Schot *et al.*, 2020).

### 1.2 Theoretical framework

The present study took a sociocultural perspective, with the assumption that knowledge is constructed in a specific social context in the relationship between individuals (Lave and Wenger, 1991). From the sociocultural perspective, the CHAT is a theory that aims to increase the understanding of how various organizational factors, such as workplace rules, the division of labor and access to adequate tools, influence the outcome of workplace activities (Engeström, 1999a, c). These factors can also interact with each other, creating contradictions within the organization. Contradictions and boundaries between professions are often viewed as obstacles to be eliminated or avoided. However, from a CHAT perspective, contradictions and diverse knowledge perspectives are crucial for learning across professions and for developing new knowledge and new working approaches. Hence, contradictions could be the driving force for necessary change and learning within an organization (Engeström, 1999a, c).

Building on the CHAT, Engeström and Sannino (2020) developed the concept of expansive learning at work, describing expansive learning as a cyclical process that aims to discover knowledge that does not yet exist. This approach suggests that learning is not inherent in professionals or organizations but emerges when they integrate, allowing new knowledge to be revealed. Expansive learning has been used in research focusing on workplace collaboration and learning, without predetermined expectations or goals for the professional’s learning process. Instead, it examines the process and its expected and unexpected outcomes based on professionals’ problem identification (Engeström and Sannino, 2020). Hence, the sociocultural perspective, using the CHAT as a starting point, could be useful when entering the IAR project and to identify and describe the project group’s improvement work and imminent processes.

## 2. Methods

### 2.1 Context and participants

The project took place in Sweden on an 18-bed ward at a medium-sized hospital (700 beds). The ward had an average hospital stay of 10 days. The clientele were elderly patients (>65 years old), most of whom receive regular care from municipal and/or primary care services when not admitted to the hospital. Most of the patients who were admitted to the ward had multiple diseases and needed extensive care.

The annual outline patient safety survey revealed deficits concerning patient transfers between wards at the hospital, cooperation with external caregivers and information transfer between working shifts. It also showed that the professionals at the ward had general difficulty completing their daily tasks related to their professional responsibilities. The ward manager and a person from the hospital’s human resources (HR) department asked for staff on the ward to voluntarily participate in a project group to work with the survey results to improve patient safety at the ward. The project group was also asked to review the daily tasks that were not followed through by the professionals to identify whether routines or working approaches needed to be changed. The professionals who applied to participate in the project group were three assistant nurses, two registered nurses, one physician, two physiotherapists and two occupational therapists. The first author was assigned as the project leader, as she was employed both as a nurse specialist on healthcare development and a researcher at the ward. At the beginning of the project, there was no established end date.

Throughout this paper, the term project group refers to the professionals in the project group, while the term ward team refers to the all the professionals working on the hospital ward.

To be able to follow the imminent bottom-up improvement processes of the project group, the IAR was assessed to be a useful approach. Being an IAR researcher (IARr) means conducting research within your own organization, as compared to when a researcher only enters the organization during a brief period to gather data (Adler and Adler, 1987). The purpose of this approach is for both the researcher and colleagues to achieve common insights within their organization (Rönnerman, 2004). The strength of action research (AR) is the focus on finding solutions to practical problems together. Such an approach promotes active participation in practical situations to improve praxis and provide significant understanding (Coughlan and Brannick, 2014; Meyer, 2000, Winter *et al.*, 2001). Within AR, the change process is followed systematically, and the researcher is continuously reflecting on the ongoing processes (Rönnerman, 2004).

### 2.2 Ethical considerations

In AR, ethics entails fostering genuine relationships between the action researcher and their peers. This involves collaboratively sharing knowledge production with all participants (Coghlan and Brannick, 2014). This meant striving for collaborative inquiry to include and encourage independent thinking from the participants and team members (Winter, 1989). Someone who is an IARr in their own organization must consider how the IAR process might benefit or harm the participants or the team members (Herr and Anderson, 2005). The present study followed the ethical requirements according to the Helsinki Declaration (World Medical Association, 2013). Unlike in other research designs, it is not possible to guarantee confidentiality within AR (Williamson and Prosser, 2002). Instead, all clinical managers (the ward manager, the managers for the physiotherapist, the occupational therapist and the head of the clinic, who was also the manager for the physician) and the participants in the project group were informed about the aim of the study and that the results would be presented without indicating names or ward connection.

### 2.3 Data collection

The project group met 11 times between 2019 and 2021. The IARr was responsible for calling the participants in the project group to the meetings, always with a copy of the e-mail to the ward manager.

The project group’s focus during the meetings was on analyzing the results from the patient safety survey and working with the identified areas that needed improvement and prioritize among areas. After this, the IARr introduced and tested the project group’s improvements at the ward and followed these improvements and working processes continuously. An action plan was the document that acted as a compass to keep the work on track and as a help when doing prioritizations in the project group. The IARr documented notes on meetings and revised and updated the document with planning/prioritization and decisions during the project group meetings. The IARr recorded the daily observations and ongoing processes within the ward between meetings and the outcomes of the implemented improvements, as well as personal reflections. These observations were subsequently discussed and analyzed during project group meetings, where the improvements were evaluated and revised from the perspectives of all involved professionals.

Due to the coronavirus disease 2019 (COVID-19) pandemic, the project group sometimes needed to postpone meetings or conduct meetings without all participants being present. In addition, during these two years, several of the participants in the project group changed. Several nurses ended their employment on the ward during these two years and during 2021 no nurses participated in the project group, except from the IARr. Hence, the IARr contributed with nursing competence at the meetings when the project group was short of a nurse representative. During 2021, a vast majority of nurses from staffing companies were hired at the ward, and these nurses had no obligation to participate in the ward’s improvement projects. Also, the physiotherapists and occupational therapists were exchanged during this period. To be able to focus on the analysis, the IARr must step out of the field (Nyman *et al*., 2015). To start the analysis, the IARr stopped participating in the project group and gathering data at the end of 2021.

### 2.4 Data analysis

After leaving the project group, the analysis and writing of the two-year project process started in the spring of 2022. Notes from the project group meetings, planning and prioritization documents, and the IARr’s own field notes were used as data for this analysis.

**Figure 1:**
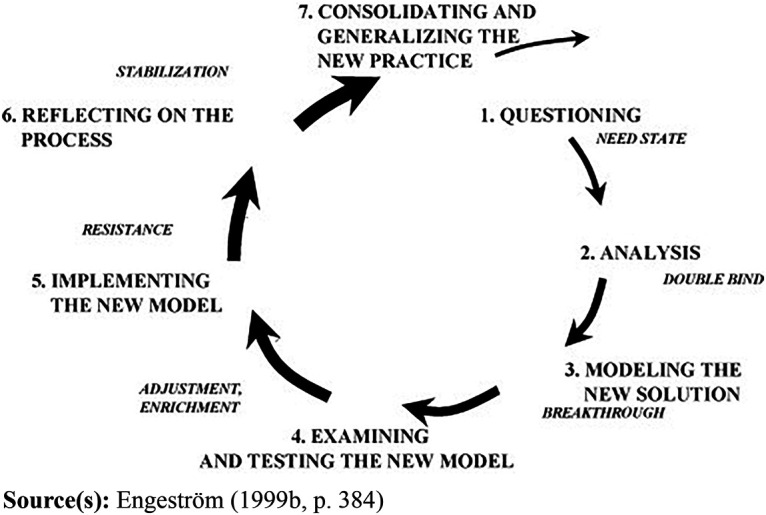
Sequence of learning actions in an expansive learning cycle

When doing the analysis, the cycle of expansive learning (Engeström and Sannino, 2020) was used as an analyzing tool. The cycle made it possible to sort and illustrate the challenges, contradictions and negotiations that had occurred during the project group’s improvement processes. The cycle of expansive learning includes seven steps: questioning, analysis, modeling a new solution, examining and testing a new model, implementing the new model, reflecting on the process, and consolidating and generalizing new practice (Figure 1, Engeström, 1999b, p. 384).

The data were sorted and written in a timeline from 2019 to 2021. Then, all data from each year were analyzed several times to place them in steps 1–7 in the cycle of expansive learning. Using these seven steps when sorting the data helped the IARr to understand the project group’s complex processes and the challenges that occurred during these two years. Making the timeline helped when sorting the data into the chronological cycle. During this initial sorting, it was determined that certain actions were more essential to the direction of the ongoing process. However, as the data were extensive, the sorting and analysis of data showed that the project group’s process did not follow the cycle’s steps chronologically; instead, the two were merged into each other. To understand which action had led to another, and why, it became necessary to analyze the data several times. This meant that some identified actions in the IAR process have been moved between steps 1–7 in the cycle several times during the analysis before finally writing the results.

## 3. Results

The results describe the process of interprofessional collaboration on a hospital ward providing safe and high-quality care. To make the process comprehensible, the results are presented in accordance with the seven steps in the expansive learning cycle (see Figure 1).

### 3.1 Questioning

The starting point of this project was the routine patient safety survey that was filled out by all staff members in the ward team. The results showed deficiency in four areas: negative perceptions related to feedback and communication about medical errors; overall security awareness; the manager’s actions regarding patient safety; and handovers and transfers of patients and patient-related information. Consequently, the patient safety survey revealed complex issues regarding the ward’s daily routine work, interprofessional communication and collaboration. In addition, the ward’s use of assistant nurses to perform nursing assignments added to the collaborative work challenges, which also needed to be examined. The project group had to address these identified areas collaboratively.

### 3.2 Analysis

The project group started to discuss the working structure at the meetings, expectations for the work, participation in the meetings and commitment to the project. The project group then started the analysis of the areas with the highest percentage of negative results in the survey, but also considering the current challenges regarding task-shifting. The work proceeded by prioritizing these areas and developing an action plan. During the analysis of the survey results, the project group identified a need to scrutinize the patients’ recovery process from admission to discharge to address as many of the critical areas identified in the patient safety survey as possible. The project group started to understand how the daily team-round routine affected the time between patients’ admission and discharges. The project group shared their views regarding the difficulties in communicating relevant information about patients’ health between professionals, working shifts and to/from external caregivers. The project group believed that these difficulties could lead to unnecessarily long admission times at the ward and, accordingly, to unsafe care. Therefore, the project group decided to prioritize the daily team-round routine at the ward and enhance the sharing of patient information associated with the team-round and the patients’ discharge process. Starting here also involved addressing the issues of unclear professional responsibilities and boundaries at the ward.

### 3.3 Modeling the new solution

When starting to model a new solution to the daily team-round routine, the members of the project group had different professional opinions about how to deal with the patients’ own active involvement in their recovery process. The physician, who had no prior experience in person-centered care on team rounds, felt that discussing the patients’ involvement in their recovery process was not a top priority at that time. However, the assistant nurses, who had recently attended a course on person-centered care, were eager to introduce this into the patients’ recovery process. When discussing the different professionals’ responsibilities and the importance of conducting person-centered care, it was evident that the project group members have different ideologies on how care should be provided from their different perspectives. The project group members realized the need to discuss which core values were valid at the clinic and how to incorporate them into the work with the patients’ recovery process. The project group believed that it was important to start here, so it spent an entire meeting phrasing the meaning and content of the clinic’s core values, which would serve as a common foundation. These core values were defined as *care on equal terms, care with the individual’s right to respect, care based on the individual’s needs,* and *care with* *a* *focus on quality and development*. Defining the content of the core values engaged the whole project group and opened a discussion about the different professionals’ beliefs and interpretations of their work. Also, the project group were given the opportunity to express and discuss their professional perspectives and responsibilities, and to find the lowest common denominator when defining the core values. The project group held a lively and constructive discussion at this point.

After the project group members had accepted the core values as valid and essential for the continuing work, the work with the team-round routine could continue. The routine was changed to start with the profession that had the latest information on the patients’ daily health status. Accordingly, the team round started with the assistant nurse’s updates about the patients’ current well-being. This was followed by the nurse adding information about the patients’ identified nursing status, needs and health risks during the hospital stay. The physician then summarized the patients’ medical status, test results, mobilization restrictions and planned examinations. Thereafter, the physiotherapists and occupational therapists informed the entire ward team about the patients’ mobilization and rehabilitation goals for the day and about how to handle the restrictions and what observations needed to be considered by the rest of the ward team. Based on the information from each professional, the round ended by deciding approximately how long the patient needed to stay at the hospital and agreeing on a date for discharge. After the team rounds every day, external caregivers were provided with information about current patients through a digital care planning system to prepare for taking over their care. The person responsible for the digital care planning system that day, typically a nurse or assistant nurse, provided the external caregivers with this information.

All of the professionals’ knowledge and competences were now integrated into the patients’ recovery process as a basis for decisions about the patients’ upcoming discharge and how they assessed the patients’ needs. This way of doing the team rounds was very different to how it had been managed before, when the assistant nurses did not participate in the rounds and the physicians, or the physiotherapists exclusively led the rounds. In parallel with the team-round work, the physician suggested that the IARr should create a digital working tool to illustrate the patients’ recovery process (see Table 1). Hence, a prototype of a digital planning board was created, illustrating the patients’ recovery process from admission to discharge. The goal of this digital planning board was to obtain a timeline that made patient care planning transparent for the entire ward team, which was deemed important for the ward team’s collaborative work. The prototype of the planning board was then presented to the rest of the project group for further decisions about layout and content.

### 3.4 Examining and testing the new model

The project group examined and tested the layout and content of the digital planning board. The ward already used a whiteboard to write down memos about every patient, such as time of shower and date of last defecation, but also decisions about whether to administer cardiopulmonary resuscitation or not. The project group agreed that the information on the whiteboard needed to be updated regarding its content and patient safety. This was an extensive debate in the project group, as several working approaches and routines had to be changed at the ward. For example, one decision that had to be made by the nurses and the physician was that all patient information, which had previously been written on the whiteboard, must be documented in the patients’ medical records and reported after a common standard between working shifts. Here, the assistant nurses had to adjust to this new way of work, as they were not used to writing in the patients' medical records. The project group finally agreed that the planning board should be used to illustrate a patient’s recovery process as a “red to green traffic sign”, where the patients’ stage of recovery determined when to move from red (newly admitted) to green (ready to be discharged). Each color on the planning board consisted of several steps that needed to be finished to move the patient’s recovery status forward. The digital planning board turned out to be a very helpful tool at the team rounds and provided an overview of how much each patient had recovered according to their individual care plan and when patients were approaching their discharge day (see Table 1).

However, new challenges arose when the project group defined the steps of the patients’ recovery process. The patients’ journey forward in the recovery process was dependent on the professional’s awareness of their own and other professions’ responsibilities and specific duties. For example, assistant nurses who were responsible for reporting to the external caregivers about the patients’ planned discharge were uncertain about what information they should report and what information the nurses and physiotherapists should provide to this report. In addition, the assistant nurses described difficulties interpreting all information in the patient’s records and handled this by writing Post-it notes to the nurses reminding them to update profession-specific information to the external caregivers. During the nurses’ and assistant nurses’ brief meetings during the working shift to discuss what needed to be done with the patients before discharge, it was revealed that the unclear professional responsibilities and boundaries had led to tasks being left undone in the belief that they were being carried out by members of another profession. This was observed when the nurse thought the assistant nurse was performing tasks and vice versa. It could also be that the same tasks were done by several professionals. For example, when the project group was planning how to prepare the patients for their discharge, it appeared that both the occupational therapist and the assistant nurse had given the same information to the patients, unaware of each other’s duties and responsibilities. These professional unclarities made the project group realize the need to discern the meaning of the professional boundaries and roles; that is, to determine which profession was responsible for certain assignments according to their professionals’ qualifications, and who was assigned daily to perform the tasks and why. It was necessary to clarify the different professional responsibilities throughout the patients’ hospital stay to ensure safety and that care was provided by the right profession according to their legislation.

When further examining and testing the digital planning board, the members of the project group agreed they when they assessed a patient’s health status to be discharge-ready, this would be marked green on the digital planning board. The members offered their own opinions about how they assessed the patients to be ready to go home. This showed how the participants in the project group had different understandings of the need to learn about each other’s professional obligations and assessments. However, the project group members learned about each other’s assessments of the patients’ health status, and which goals needed to be fulfilled to discharge a patient safely (see Table 2). The job involved a lot of meetings, where one professional’s goals and responsibilities were scrutinized at a time. It was more difficult for occupational therapists and physiotherapists to reach a consensus about when and how to phrase the “discharge-ready” goals according to their professional obligations. The occupational therapists and physiotherapists sometimes wanted to keep the patients admitted for longer because they were uncertain whether the patients would receive the necessary care after discharge due to the municipality’s lack of resources.

The physiotherapist and occupational therapist assessed the patient as being discharge-ready by observing their physical functions, such as their movement pattern or need for assistive technology or aids. The physician determined that the patients were discharge-ready based on a medical perspective, and their care needs could end once the test results were acceptable, and the patient was symptom-free. The nurses’ assessments were made based on a specific nursing model for assessing patients' health status. This stage in the discussion about the different perspectives on a discharge-ready patient finally resulted in written professional goals. Consequently, the process of developing the digital planning board led to a revised illustration of patients as discharge-ready according to the set goals from medical, nursing, physiotherapy and occupational therapy perspectives (see Table 2).

### 3.5 Implementing the new model

The new daily team-round routine was (permanently) introduced to the ward team. The structure of the team round helped to follow the agenda and set time for the rounds. The project group noted that the new structure revealed the different professional responsibilities and what information each profession was responsible for sharing. The project group and the ward team saw how the new round routine helped them to collaborate and communicate better when it was evident what questions were to be discussed, which profession should be present, and what responsibilities were on the set agenda. The ward team considered that the digital planning board clarified the daily tasks and reduced the risk of misunderstandings. The daily morning meetings provided the ward manager with information about how far each patient’s recovery process had progressed, enabling him/her to calculate the ward’s bed occupancy. Better collaboration and communication between shifts were another positive effect recognized by the ward team, as planning for patient discharge could begin during the earlier shift.

### 3.6 Reflecting on the process

According to the project group, the ward team initially collaborated better when the team round had a clear aim and agenda, and all professionals knew their professional responsibilities at the team rounds. The digital planning board exposed daily assignments and improved the flow and time between patients’ admission to discharge. This overall structure around the discharge process also contributed to the ward’s efficiency, as the discharge process was not unnecessarily delayed. However, after the digital planning board had been implemented and the goals for being discharge-ready had been defined, disagreements and frustrations started to arise in the ward team and in the project group. The ward team had disagreements about when patients were ready to be discharged, which caused collaboration difficulties. The occupational therapists and physiotherapists took more time to prepare the patients for discharge, and the team began to doubt the different requirements for discharge readiness.

At this point in the process, the project group struggled to determine which profession would receive the most interpretation priority due to the diverse perspectives regarding being discharge-ready. Due to the collaborative challenges in the project group, two clinical managers (the head of the clinic and the physiotherapists’ manager) participated in a meeting to address the lack of clarity related to the process of being discharge-ready. The clinical managers helped clarify the boundaries between the various responsibilities of the caregivers; that is, the hospital and the municipality. A common understanding among the managers was that the different professionals in the ward team were not assessing patients to be discharge-ready in the same way or at the same speed. According to the assistant nurses, the physiotherapists in the ward team were neglecting their skills and assessments of the patients, which led to patients being admitted for a longer time than necessary. The physiotherapists considered the agreed goals for being discharge-ready to be adequate but expressed that they were not given the prerequisites, such as necessary time with the patients, to reach the set goals within a reasonable time. Frustration arose during this meeting between the assistant nurses and the physiotherapists, but also between the head of the clinic and the professionals. The head of the clinic expressed the importance of discharging patients more rapidly, while the assistant nurses expressed feelings of being ignored by the physiotherapists in their competence to care for the patients’ daily activities to help speed up the discharge process. This was a turbulent meeting that involved frustration, disagreements and raised voices between the participants. Motivating the project group members to stay and work on solutions for these issues was a challenge following this meeting.

However, the project group gathered for the subsequent planned meetings. The project group discussed the goals for being discharge-ready and what was hindering their work. During this brainstorming session, the IARr took notes that everyone could see, revealing the reasons for the frustrations and disagreements. The project group had to clarify the goals for being discharge-ready again, as they saw that the professional boundaries were still unclear in the ward team. A mind map created during the brainstorming session showed how task-shifting had forced assistant nurses to perform tasks for all other professionals without having their responsibilities clarified or communicated.

Therefore, the process continued to determine when and by whom the medical, nursing, physiotherapy and occupational therapy goals could be reached. The purpose of clarifying each goal was to find ways of collaborating in the ward team to grasp the professional responsibilities and limitations in relation to each other and to be able to share the different tasks related to the patients’ recovery process. This was considered the key to resolving disagreements and frustration (see Table 3).

### 3.7 Consolidating and generalizing the new practice

The new team-round routine and the digital planning board had been in operation for six months when the IARr ended the data collection in 2021. Despite plans to follow-up on the project during the autumn of 2021 with the same routine patient safety survey, the survey was withdrawn due to organizational changes at the clinic. However, the ward manager reported that the number of medical errors concerning patient transfers and discharge had decreased slightly after the new routines were implemented.

The project group discussed the continuation of further developing the routines and shared their knowledge with the ward team and other clinical managers. For example, the nursing goals were largely dependent on the assistant nurses’ reports of patients’ status to the nurses because the nurses often needed to prioritize administrative tasks over bedside care. However, the medical, physiotherapy, and occupational therapy goals were dependent on the assistant nurses providing them with information about the patients’ health and the results of performed assignments.

Once the digital planning board was set and the team-round routine was functioning, the next step was to illustrate the recovery process map for the patients, providing them with the opportunity to be an active part of their own recovery process. The project group drew up suggestions for an information sheet and brochures to give to the patients when they were admitted. The progress of this process is still unclear due to the organizational changes at the clinic.

## 4. Discussion

The aim of this study was to identify and describe the collaborative and professional boundary challenges at a hospital ward from a bottom-up perspective. The greatest recurring challenge, which caused contradictions and hampered the collaborative work in the project group, was unclear professional boundaries; that is, the unclear professional responsibilities and distribution of tasks between the professions. Due to frequent staff turnover, especially among nurses, the professional boundaries had become more unclear on the ward, where assignments were performed by available staff and not always by the formally responsible professional. The lack of boundary clarity can have consequences concerning patient safety and collaboration (Morley and Cashell, 2017), while initiatives that soften or break down boundaries in healthcare teams can hinder interprofessional collaboration (Farchi *et al.*, 2023). The results of the present study confirm previous study results, which identified the importance of clarifying the professional boundaries that largely impacted the daily work at the hospital ward, and hence, the outcomes of healthcare quality and safety, which requires negotiation and clarification of professional boundaries (Schot *et al.*, 2020).

When scarce resources demand heightened flexibility in professional roles to manage daily tasks, Nancarrow and Borthwick (2005) caution against potential conflicts among professionals. They highlighted an increased risk of disputes when tasks are carried out by professionals from diverse disciplines possessing similar levels of training and expertise compared to when tasks are performed over professional boundaries that are not equivalent (Nancarrow and Borthwick, 2005). The results from the present study also show that disputes arose between the assistant nurses and physiotherapists in the project group as they shared several practical tasks at the ward without having discussed who had the professional responsibility for the task according to their discipline (Nancarrow and Borthwick, 2005; Richards *et al.*, 2000). Our results show that the most difficult professional boundaries to define were those between the assistant nurses and the other professionals in the ward team. The assistant nurses were involved in several of the other profession’s daily tasks, without having clarified the boundaries of the tasks. Being able to assess the patients as discharge-ready, the project group understood the need to organize the daily work to support the different discipline’s legal responsibilities in relation to the patients’ recovery process.

Therefore, the project group needed to learn about each other’s professional obligations and ethical codes according to each discipline. The profession’s different ethical codes, professional values, and legal responsibilities illustrated the complexity of the ward team’s collaborative work. Hence, to agree on common goals and values, beyond their profession-specific ones, was a prerequisite for a sufficient collaboration within the team, as also described by Patel *et al.* (2012). The understanding of the common core values could also be significant to reduce power relations within a group, as the group members’ education and status could affect whose knowledge should be the most respected in the group (Richards *et al.*, 2000). When conducting this project, the power structures in the interprofessional ward team were demonstrated and are, according to Hutchings and Jarvis (2012), commonly related to status or the perceived right to claim superiority of interpretation in different contexts. In this study, the turbulent meeting described in the results section provides an example of how status and superiority of interpretation caused disputes and tensions. As the assistant nurses had the lowest level of formal education in the project group, they were frustrated about not being valued as a profession. Similarly, the head of the clinic, who was present at the meeting, was putting pressure on the physiotherapists to make discharge decisions more rapidly due to the increasing need for care at the ward. Consequently, the physiotherapists were required to speed up their assessments and training of the patients to be able to make discharge-ready decisions as quickly as possible. They were unable to perform their work satisfactorily according to their professional responsibilities and values.

Accordingly, contradictions related to hierarchy, internal power structures and educational background were uncovered during the meeting. Professional values and established goals for determining discharge readiness had to be negotiated within the project group, where the precedence of interpretation was clearly influenced by hierarchical positions and levels of education. As described by Andersson and Lindström (2017), joint reflections between the professionals could enhance boundary awareness on professional-specific priorities and identify inter-organizational tensions. Also, Akkerman and Bakker (2011) discussed how boundaries between professionals have an ambiguous nature, as they can trigger negotiations and be challenging, while at the same time, boundaries could be meaningful learning opportunities for professionals (Akkerman and Bakker, 2011).

The results presented in the present paper highlight the need to integrate the diverse competencies of various professions to assess a patient’s readiness for discharge. The findings indicate that the ward team members need a better understanding of each other’s professional competencies and responsibilities. This study suggests that enhanced understanding and trust in each other’s professional roles could reduce interprofessional tensions. The lack of knowledge about other professions’ education and competencies not only complicates the identification of professional boundaries in daily practice but also hampers effective collaboration.

## 5. Methodological considerations

Conducting IAR in one’s own organization brings several methodological reflections and challenges. Both strengths and limitations of the IAR process can be identified. The strengths of the present study are the researcher’s active and long-standing participation in the project as a senior nurse. However, the IARr’s situation can also be problematic due to the occasional difficulties of maintaining the necessary distance. The IARr ended the data collection several months before starting the analysis process to gain distance from the process, which is necessary in order to begin the final write-up of the manuscript, as described by Nyman *et al.* (2015). Another challenge when conducting IAR is that the ongoing process can create and reveal underlying conflicts and frustration that the IARr must be prepared to manage (Meyer, 1993). As described in the ethical considerations, the IAR’s own values and norms have been the compass to assess and manage these dilemmas. Additionally, the IARr has had continuous discussions with the research team outside the hospital to reflect on her own preunderstanding and to address contradictions in the project group.

Using the cycle of expansive learning for the analysis revealed interprofessional contradictions and outcomes that had not been expected when the project started, as described by Engeström and Sannino (2020). These contradictions did not become noticeable until the new working models were tested and implemented during the IAR process. For example, when starting up this process, neither the project group nor the IARr knew which measures would be decisive to move the process, or why certain challenges became more important to manage than others. What was deemed important was allowing the ongoing process to determine the teams’ learning needs and outcomes (Engeström and Sannino, 2020). Similarly, as described in AR, participants translate knowledge into action through critical reflection and learning (Koch and Kralik, 2006). Furthermore, incorporating the IAR perspective during the process created an opportunity to allow the diverse professionals’ learning and insights to shape the direction of development and the implementation of the new team round. Hence, collaborative work in another context might be described differently than how it will be described in this project because the working outcomes are believed to be dependent on personal engagement, organizational prerequisites and social interactions within the workplace (Engeström and Pyörälä, 2021).

Ongoing staff turnover led to frequent changes in professional representation within the project group over the two-year period. The IARr assumed the role of representing the nurses’ perspective at the nursing meetings and conveyed their opinions to the project group. The assistant nurses were represented by three participants in the project group, but only one could participate at a time. However, the assistant nurses had varying responsibilities, which resulted in the absence of certain specific expertise during project group meetings. Similarly, the occupational therapists rotated participants throughout the process, remaining active and informing their colleagues between meetings. Initially, the physiotherapists were effective with two participants in the project group, but there were changes in participation among physiotherapists in the later meetings. However, there were changes in terms of who participated in the project group during the last meetings. The IARr, the physician and the assistant nurses remained constant participants throughout the project. The frequent staff turnover highlights the challenges of maintaining continuity in collaborative work, particularly among nurses (Liang *et al.*, 2018; Gustavsson *et al.*, 2020).

## 6. Conclusion

This study aimed to identify and describe the collaborative and professional boundary challenges at a hospital ward from a bottom-up perspective. The findings highlighted that the primary challenge hindering effective collaboration was unclear professional boundaries, which were exacerbated by frequent staff turnover. These ambiguities in responsibilities and task distribution, particularly between assistant nurses and collaborating professionals, led to conflicts and potential risks to patient safety. The study highlights the importance of negotiating and clarifying professional boundaries to support interprofessional collaboration, which is necessary for delivering safe care with limited resources. Establishing a shared understanding of professional roles, ethical codes and common goals appears to be essential for reducing internal power dynamics and enhancing teamwork. Consequently, additional efforts are needed to address these boundary issues and the consequences of constructing, blurring or breaking boundaries down while introducing new ways of working, as they might influence the safety of healthcare delivery.

## 7. Implications

The results from this study identified a future need to explore how hospital care could be safer by clarifying boundaries, without jeopardizing patient safety and interprofessional collaboration. In this IAR project, the social order in the project group, and consequently the ward team, was challenged and changed. When the project group learned about each other’s competences and professional responsibilities, the round routine was revised. The new team-round routine enhanced the assistant nurses’ status and importance in the ward team as being the first profession on the agenda for the team round. The physician, who had previously made all decisions at the team round, learned to consider all professional assessments and knowledge about the patients. However, when meeting the head of the clinic, it became clear that this new agenda and challenged hierarchical order at the team rounds were not generally accepted or willingly supported; instead, the new way was that the team round was believed to prolong the patients’ stay at the ward. This study has shown how it is difficult to challenge hierarchical orders. Also, interprofessional collaboration is hampered daily by the lack of staff, where professional boundaries are blurred and ignored. Making a patient discharge-ready from all professional perspectives could, from a long-term perspective, make patient discharges safer, and it could also contribute to more efficient care as readmissions could decrease.

## Figures and Tables

**Table 1:** Illustration of the patients’ recovery process from admission to discharge

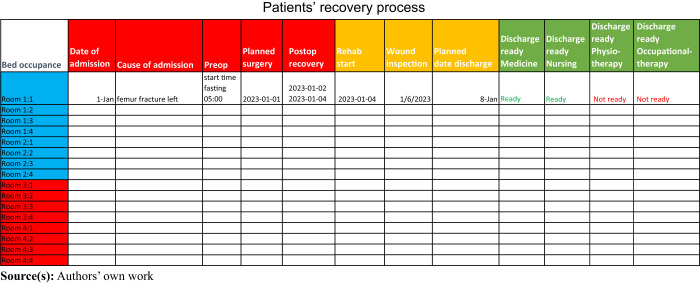

**Table 2:** Example of goals for discharge-ready from medical, nursing, physiotherapy, and occupational perspectives

Goals medicine	Goals physiotherapy	Goals occupational therapy	Goals nursing
The infection markers are decreasing	The patient must have a functioning locomotion	Assessment of the need for occupational therapy must be done and documented in the patient’s journal	Nursing actions have been started based on what is reasonable to achieve during the hospital stay
The hemoglobin level is increasing	The patient should have as independent locomotion as possible in relation to previous abilities and current preconditions	The patient should become as independent as possible in their everyday activities of daily living. A report is done to external caregivers according to the continued plan for everyday activities in daily life and the need of aid	A plan for the follow-up for the nursing actions after discharge must be ready
Afebrile	There should be an assessment and a plan for the external follow-up. The digital planning system for external caregivers must be updated	Adequate aid is safely prescribed or reported to external caregivers for follow-up	The patient and relatives must be informed and involved during the discharge process
The wound heals as expected			The nurses have the final responsibility to certain that the patients have all necessary papers, information, and drugs when being discharged
Test results and vital signs normalizes (expected) or are within the patient’s normal range. Not acute, remaining pathology, is referred further			
The patient is symptom-free			
The pharmaceutical prescription is updated			

**Source(s):** Authors’ own work

**Table 3:** Example of how the medical goals for discharge-ready needed to be scrutinized according to how, when and by whom

Goals medicine	How?	When?	By whom?
The infection markers are decreasing	Regular/routine blood sampling	According to routine or after the decision at the team round	Nurse
The hemoglobin level is increasing	Regular/routine blood sampling	According to routine or after the decision at the team round	Nurse/assistant nurse
Afebrile	Controls according to routine or if necessary	According to routine or after the decision at the team round	Nurse/assistant nurse
The wound heals as expected	Daily reconciliation with patients and staff	Follow-up at team round	Patient/nurse
Test results and vital signs normalize (expected) or are within the patient’s normal range. Not acute, remaining pathology, is referred further	Controls according to routine	Follow-up at team round, controls according to routine or after assessment	Patient/nurse → external caregivers
The patient is symptom-free	Review of the patients' journal, test results, reconciliation/discussion with staff. If necessary, also reconciliation with relatives/staff in the municipality for information on habitual status	Continuously during the hospital stay, especially when approaching the date for discharge	Nurse/patient/relatives → external caregivers
The pharmaceutical prescription is updated	Review of the patient’s drugs before admission. Reconcile the current drug list with the patients' drugs before admission in the last 6 months/update the medical journal system	Continuously during the hospital stay, especially when approaching the date of discharge-ready	Physician/pharmacist

**Source(s):** Authors’ own work
